# Artificial intelligence-assisted cryoEM structure of Bfr2-Lcp5 complex observed in the yeast small subunit processome

**DOI:** 10.1038/s42003-022-03500-y

**Published:** 2022-06-01

**Authors:** Yu Zhao, Jay Rai, Chong Xu, Huan He, Hong Li

**Affiliations:** 1 Institute of Molecular Biophysics, Florida State University, Tallahassee, FL 32306 USA; 2 Department of Chemistry and Biochemistry, Florida State University, Tallahassee, FL 32306 USA

**Keywords:** Cryoelectron microscopy, Ribosomal proteins

## Abstract

Eukaryotic ribosome is maturated through an elaborate process that includes modification, processing and folding of pre-ribosomal RNA (pre-rRNAs) by a series of ribosome assembly intermediates. More than 70 factors participate in the dynamic assembly and disassembly of the small subunit processome (90S) inside nucleolus, leading to the early maturation of small subunit. The 5’ domain of the 18S rRNA is the last to be incorporated into the stable 90S prior to the cleavage of pre-rRNA at the A1 site. This step is facilitated by the Kre33-Enp2-Bfr2-Lcp5 protein module with the participation of the DEAD-box protein Dbp4. Though structures of Kre33 and Enp2 have been modeled in previously observed 90S structures, that of Bfr2-Lcp5 complex remains unavailable. Here, we report an AlphaFold-assisted structure determination of the Bfr2-Lcp5 complex captured in a 3.99 Å − 7.24 Å cryoEM structure of 90S isolated from yeast cells depleted of Pih1, a chaperone protein of the 90S core assembly. The structure model is consistent with the protein-protein interaction results and the secondary structures of recombinant Bfr2 and Bfr2-Lcp5 complex obtained by Circular Dichroism. The Bfr2-Lcp5 complex interaction mimics that of exosome factors Rrp6-Rrp47 and acts to regulate 90S transitions.

## Abstract (web summary)

AlphaFold-assisted CryoEM structure determination reveals the Bfr2-Lcp5 complex structure in the yeast 90S pre-ribosome.

## Introduction

Eukaryotic cells employ an elaborative process to produce appropriately assembled ribosome. This process is initiated in the nucleolus by transcription of a single 35S ribosomal RNA precursor (pre-rRNA) that encompasses three rRNAs (5.8S, 18S and 25S) and the 5’ and 3’ externally or internally transcribed spacer regions (5’-ETS, 3’-ETS, ITS1, and ITS2)^1–3^. Pre-rRNA is then chemically modified by over one hundred small ribonucleoprotein particles (snoRNPs) and processed by the small subunit (SSU) processome (90S) into separate rRNAs with the spacer regions removed. This process is concomitant with and followed by assembly of ribosomal proteins to form the early mature intermediates of subunits that are then transported to cytoplasm for further maturation.

A key to understand ribosome biogenesis is to elucidate the structure and function of the early acting 90S complex. Nearly 70 non-ribosomal factors^2^ along with some ribosomal proteins^4^ associate with the pre-rRNA to form 90S, many of whom are implemented in diseases^5^. Powered by several ATPases and seemingly unknown molecular switches, 90S successively compacts and processes pre-rRNA to allow removal of the spacers^6–11^. Recent biochemical and structural studies of the yeast and human 90S have begun to delineate the functional role of the biogenesis factors in this highly dynamic process. Remarkably, the compositional and conformational changes in 90S appear to be dictated by specific interactions among the 90S factors and with the pre-rRNA^12–14^. Structurally defining these interactions along the functional pathway of 90S, therefore, is an essential aspect in studies of ribosome biogenesis.

The Kre33 module, a subcomplex comprised of Kre33, Enp2, Bfr2 and Lcp5 in yeast, has been shown to act as the last element to place the 5’ domain of 18S^11^. When bound in 90S, the Kre33 module is observed to hold in place several elements of the pre-rRNA and the 5’ ETS in the assembly phase of 90S. Following or concomitant with cleavage of the pre-rRNA, 90S disassembles by sequentially releasing the assembly factors. The Kre33 module is released following the departure of Krr1-Faf1, which triggers a large re-arrangement of the RNA elements required for further processing and 5’ ETS degradation^6,7^. The exact mode of Kre33 module release differs in the Dhr1-depleted 90S from the Noc4-Dhr1 split-tag purified 90S. While the four members of the Kre33 module depart as a whole in the Dhr1-depleted 90S^6^, Lcp5 is first released followed by the other three members in the Noc4-Dhr1 purified 90S^7^. Interestingly, the DEAD-box protein Dbp4 was previously found to interact with Bfr2 and is believed to facilitate the association of the Kre33 module with 90S^15^. Despite the important roles, the Kre33 module is only partially resolved in all previously resolved 90S structures. Although the Kre33-Enp2 subcomplex, the short C-terminal tail of Bfr2^11^ and the C-terminal domain of Lcp5 are modeled^8,10,16^, the interface between Brf2 and Lcp5 has not been observed, suggesting that they form transiently in 90S assembly.

We hypothesized that removal of chaperones for 90S assembly can potentially alter its dynamics and thus allow the capture of transiently associated factors. At the core of the 90S assembly is the U3 small nucleoprotein particle (snoRNP). The yeast U3 snoRNP belongs to the box C/D family of snoRNPs that contain four conserved proteins: Nop1 (fibrillarin), Nop56, Nop58 and Snu13^17,18^. These proteins are typically unstable on their own and require the chaperone function of the Hsp90/R2TP system^19^. R2TP is a four-protein complex comprised of two AAA + ATPases Rvb1 and Rvb2, Tah1 (YCR060W), and Pih1 (YHR034C). R2TP has been shown to interact with a number of ribonucleoprotein particles (RNPs) including the box C/D snoRNPs that form the core of 90S^20–24^ and in its absence, pre-mature rRNA intermediate accumulate as polysomes diminish^20^. We thus determined a cryoEM structure of 90S isolated from yeast strain where Pih1 is depleted (Δ*pih1*−90S) at resolution 3.99–7.24 Å. Though the structure of Δ*pih1*-90S is highly similar to that of 90S from the wild-type cells determined under a similar cell growth and purification condition^10^, the Δ*pih1*-90S allowed us to solve the structure of the Bfr2-Lcp5 complex at a medium local resolution (~5.04 Å) aided by AlphaFold modeling^25^ and by in vitro characterizations. Our structure provides the first near atomic model of the yeast Bfr2-Lcp5 complex that is also consistent with the mapped protein–protein interactions between their human homologs, AATF and NGD^26^ and thus, suggests a conserved mechanism of Kre33 module function in 90S transitions.

## Results

### Overview of the Δpih1-90S structure

**Fig. 1:**
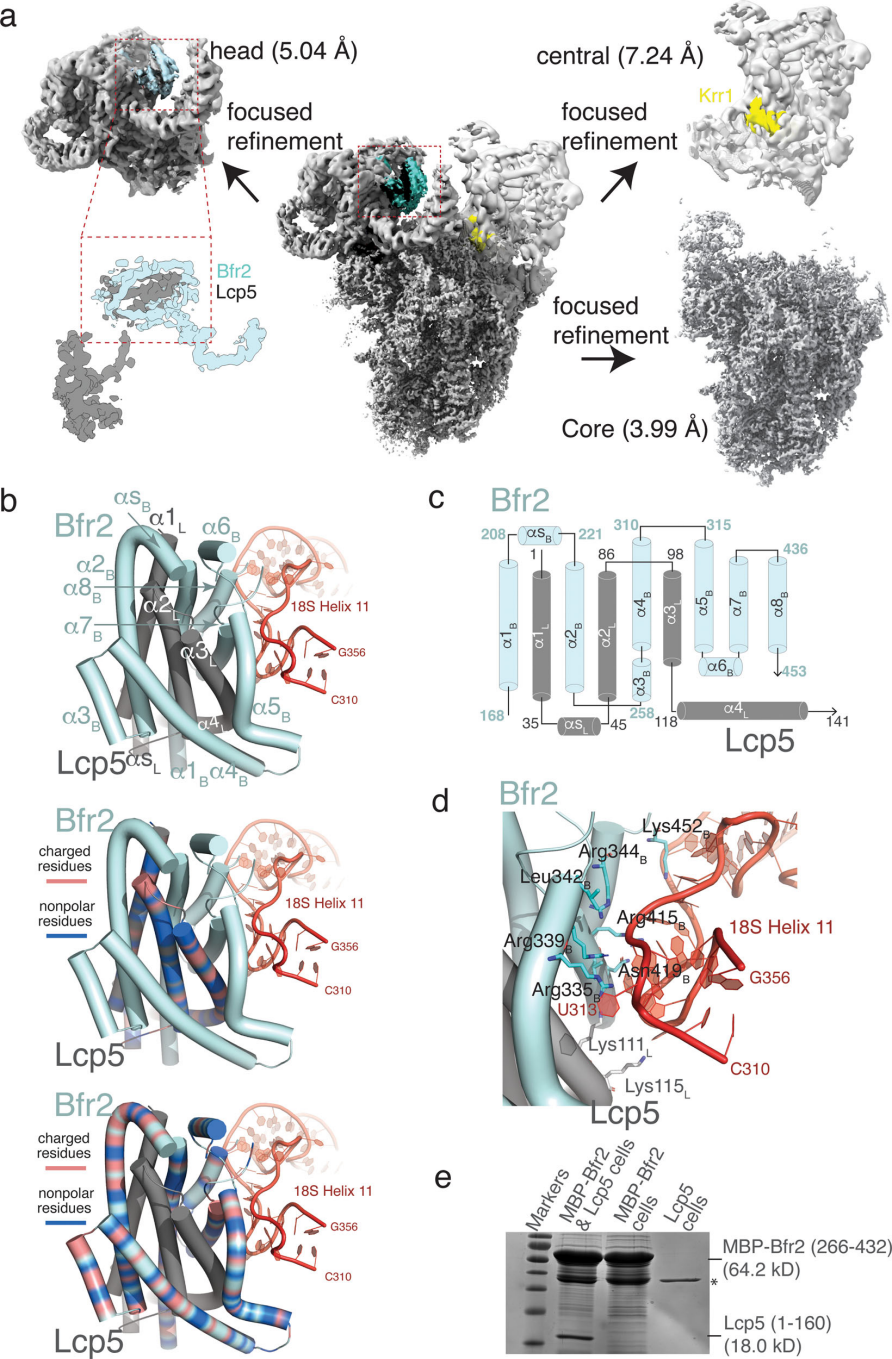
The overview of Δ*pih1*-90S structure and model of Bfr2-Lcp5 complex. **a** Reconstruction of the Δ*pih1*-90S complex displayed as electron potential contours. The core structure and two focused refined regions are shown in separate panels with their respective resolutions. The unmodeled region is highlighted in cyan in the head (5’ domain) region. The location of Krr1 is indicated by yellow. The close-up view displays density identified for the Bfr2 central domain (cyan) and Lcp5 N-terminal domain (gray). **b** Final models of Bfr2 and Lcp5. Top, Bfr2 (cyan) and Lcp5 (gray) are shown in cartoons with helices represented as cylinders. The pre-rRNA are colored in red. Helices are labeled sequentially with subscript “B” representing Bfr2 and “L” representing Lcp5, respectively. “αS” denotes the short α-helix between α1 and α2 for both Bfr2 and Lcp5. Middle, in the same orientation as that on top, charged residues of Lcp5 are colored in orange and nonpolar residues of Lcp5 are colored in blue. Bottom, in the same orientation as that on top, charged residues of Bfr2 are colored in orange and nonpolar residues of Bfr2 are colored in blue. **c** Topology of Bfr2 (cyan) and that of Lcp5 (gray). **d** Predicted interactions between Bfr2-Lcp5 complex and the pre-rRNA. Residues are labeled with subscript “B” representing Bfr2 and “L” representing Lcp5, respectively. **e** SDS-PAGE analysis of pull-down assay with MBP-Bfr2(266–432) and Lcp5(1–160) on an amylose agarose column. Protein bands identified as either MBP-Bfr2(266-432) and Lcp5(1–160) are marked. A non-specific protein band is labeled by an asterisk.

We have previously characterized the growth phenotype of Δ*pih1* strain^27^. The Δ*pih1* strain exhibited slow growth at non-permissive temperatures, similar to that observed in previous studies^20,22^, and showed synthetic lethality with C-terminally deleted Nop58, a core 90S assembly factor^27^. Here we performed single particle reconstruction of 90S isolated from the Δ*pih1* strain grown to a high optical density (Materials and Methods). The genetically modified yeast strain contains TAP tagged Pwp2p (Pwp2-TAP) and a Twin-strep-tag tagged Kre33 (Kre33-SII), which allowed purification of the Δ*pih1*-90S to homogeneity by tandem affinity using the IgG Sepharose and Strep-Tactin columns (Supplementary Data 1). Data collection and processing led to a final of 199,534 particles with good quality that after reconstruction and refinement, resulted in a 3D core structure with an overall resolution of 3.99 Å estimated based on the Fourier Shell Correlation (FSC) at 0.143 (Table 1 & Supplementary Figs. 1, 2). To reduce the impact of heterogeneity on refinement, especially around the two protruding domains, we also performed focused refinement to yield 5.04 Å and 7.24 Å for the 5’ domain and the central domain, respectively (Table 1, Fig. 1a and Supplementary Figs. 1, 2).

**Table 1:** Data Acquisition, Processing and Model Refinement.

Data acquisition & processing parameters	∆*pih1*-90S
Microscope	Titan Krios
Detector	Gatan K3
Voltage	300 kV
Electron source	Field Emission Gun
Collecting mode	Super-resolution
Dose Rate (e^-^/Å^2^)	40
Defocus range (μm)	−1.3 to −2.5
Nominal magnification	81,000X
Frames collected per exposure	50
Energy filter	20 eV
Framealingment software	MotionCor2
CTF parameter estimation	Gctf
Total number of raw images collected	4644
Number of images used for particle picking	3477
Initial particles picked	233,968
2D classification software	Relion 3.1
Final reconstruction software	Relion 3.1
Applied symmetry	C1
Number of particles contributed for the final reconstruction	199,534 (Overall and central domain)
	84,570 (Head domain)
Resolution method	FSC 0.143 cut-off
Map resolution (Å)	3.99 Å (Overall)
	5.04 Å (Head)
	7.24 Å (Central)
Local resolution determining software	Resmap
Map visualization software	Pymol/Chimera/Chimera X/ Coot
Deposit EMDB codes	25,441
Refinement parameters	
CC (map_model)	0.77 (mask), 0.77 (volume), 0.83 (box)
RMSD (Bond lengths/Bond angles)	0.008/0.719
Ramachandran plot (%) (Outlier/Allowed/Favored)	(0.03/8.57/91.40)
Cβ Outliers (%)	0.00
MolProbity score	2.58
Clash score	39.08
Rotamer outliers (%)	0.18
ADP (B-factor) (Å^2^)	213241
Protein (min/max/mean)	28.46/292.48/95.52
Nucleotide (min/max/mean)	45.90/488.62/166.35
Ligand (min/max/mean)	55.90/172.44/108.55
dFSC model (0.5)	4.00
Deposit PDB codes	7SUK

The Δ*pih1*-90S complex purified by the Pwp2-Kre33 split-tag shares a strong structure homology with that isolated from the wild-type yeast (PDB ID: 5WLC), also grown under a nutrient limited condition^10^. This state of 90S is believed to be a product of 90S quality control and unable to progress further. It was, nonetheless, one of the landmark structures that unveiled the architecture of 90S^9,10^ and is believed to be reversable to those that are successive^6,7^.

Following placement of the protein and RNA models into the Δ*pih1*-90S density, it became immediately clear that in Δ*pih1*-90S, the region where C-terminal tail of Krr1 is in state B2 contains a much larger density that cannot be assigned to Krr1 (Fig. 1a and Supplementary Fig. 3a). As described immediately below, this density is identified and modeled as the complex of Bfr2 and Lcp5 N-terminal domain.

### A structural model of the Bfr2-Lcp5 complex

The density observed near helix 11 (h11) of the 5’ domain continues from the density previously assigned to the C-terminal tail of Bfr2 (residues 454–500), which immediately suggests that the density may be attributed to Bfr2. Though not at high enough resolution for protein tracing, the density exhibits clear features corresponding to several tightly packed helices (Fig. 1b and Supplementary Fig. 3a). To facilitate placement of Bfr2 into the density, we first obtained a computationally predicted model of Bfr2 by use of the machine learning-based AlphaFold^24^. The AlphaFold-generated Bfr2 comprises of 10 major helices with the low complexity regions 1–140 and 345–403 unstructured. Interestingly, while the two C-terminal helices have previously been modeled and can fit the density of the C-terminal tail, the remaining eight can be directly placed into the rest of the density with only minor adjustment in orientations. However, this Bfr2 model is insufficient to account for all the density. The density unaccounted for exhibits features that can accommodate four additional helices following Bfr2 placement (Supplementary Fig. 3b).

Bfr2 was previously found to interact with Lcp5, especially in the nutrient-limited 90S^10^, suggesting the possibility for a Bfr2-Lcp5 complex. By taking advantage the ability of AlphaFold to predict protein–protein complex structures^28^, we modeled that for the Bfr2-Lcp5 complex. Interestingly, AlphaFold resulted in an intertwined structure of the Bfr2-Lcp5 complex with Bfr2 maintaining the same fold as that in isolation and three helices of Lcp5 inserted into the Bfr2 fold (Supplementary Fig. 3b). This predicted Bfr2-Lcp5 complex model was also predicted by the method that combines AlphaFold and RoseTTAfold, taking into the consideration of co-evolution^29^. Satisfactorily, the AlphaFold-predicted Bfr2-Lcp5 complex can be readily placed into the density with only minor adjustment (Fig. 1b and Supplementary Fig. 3a–c). The final model of the Bfr2-Lcp5 complex contains Bfr2 residues 168–453 and Lcp5 residues 1–141, both with density correlation coefficient of 0.6.

Bfr2 and Lcp5 each contributes slightly more than 100 residues to formation of their interface that buries a large solvent accessible area (4843 Å^2^). Significant portion of the buried surface comprises of hydrophobic residues (83% of interface residues from either protein) (Fig. 1b), suggesting that the Bfr2-Lcp5 interface is strong. The major interaction between Bfr2 and Lcp5 arises from an analogous long-short-long helix fold, or the UTP3/SAS19 domain, in both proteins (α1-αS-α2 where “S” denotes “short”) interdigitating each other with a pseudo-2-fold symmetry (Fig. 1c). This interaction resembles that found in the complex of exosome-associated exoribonuclease, Rrp6, and its binding factor, Rrp47^30^.

The additional helices of Bfr2 (α5-α8) and Lcp5 (α3-α4), on one side, buttress against the central interdigitated Bfr2-Lcp5 helical bundle and on another, against helix 11 (h11) of 18 S RNA (Fig. 1d). The Bfr2-Lcp5 complex establishes a number of electrostatic interactions with h11 via mostly arginine and lysine residues (Fig. 1d).

To provide experimental evidence for the cryoEM model of Bfr2-Lcp5 complex, we expressed and purified Maltose Binding Protein (MBP)-fused Bfr2 fragments, untagged Lcp5 fragment 1–160 and polyhistidine tagged Lcp5 fragment 1–160 in an bacteria expression host (Supplementary Fig. 3d). The full-length or fragments including sequences prior to 175 of Bfr2 showed limited or no expression while fragments 266–432, 300–534, and 360–432 had good solubility (Supplementary Fig. 3d). We used the MBP-Bfr2(266–432) fragment in performing a pull-down assay with untagged Lcp5(1–160) in cell and MBP, MBP-Bfr2(360–432) and the MBP-Bfr2(266–432) with purified His-Lcp5(1–160) in vitro. The pull-down assay showed that MBP-Bfr2(266–432) can interact with Lcp5(1–160) but not the null control in cell (Fig. 1e). Consistently, purified MBP-Bfr2(266–432) could pull down purified His-Lcp5(1–160) while MBP or MBP-Bfr2(360–432) did not (Supplementary Fig. 4), thereby supporting the structural model of Bfr2-Lcp5. We believe that the non-stoichiometric co-elution is a result of both MBP-Bfr2(266–432) and Lcp5(1–160) expressed in bacteria forming aggregates or self-oligomers (Supplementary Fig. 4).

Bfr2 and Lcp5 share significant sequence similarity to their human homologs, AATF (34%) and NGDN (29%), respectively, in their UTP3/SAS19 domain (Supplementary Fig. 5). Previously, through affinity tag pulldown of transiently expressed AATF and NGDN in HEK293 cells, Bammert et al. showed that the UTP3/SAS19 domain, 1–148 of NGDN, was sufficient to precipitate AATF^26^. The same conclusion was further confirmed using recombinantly expressed AATF and NGDN fragments^26^. These in vitro studies are satisfactorily in agreement with our Bfr2-Lcp5 model and thus suggestive of an evolutionarily conserved mechanism of this protein complex.

### Circular dichroism studies of Bfr2 and Lcp5 fragments

**Fig. 2:**
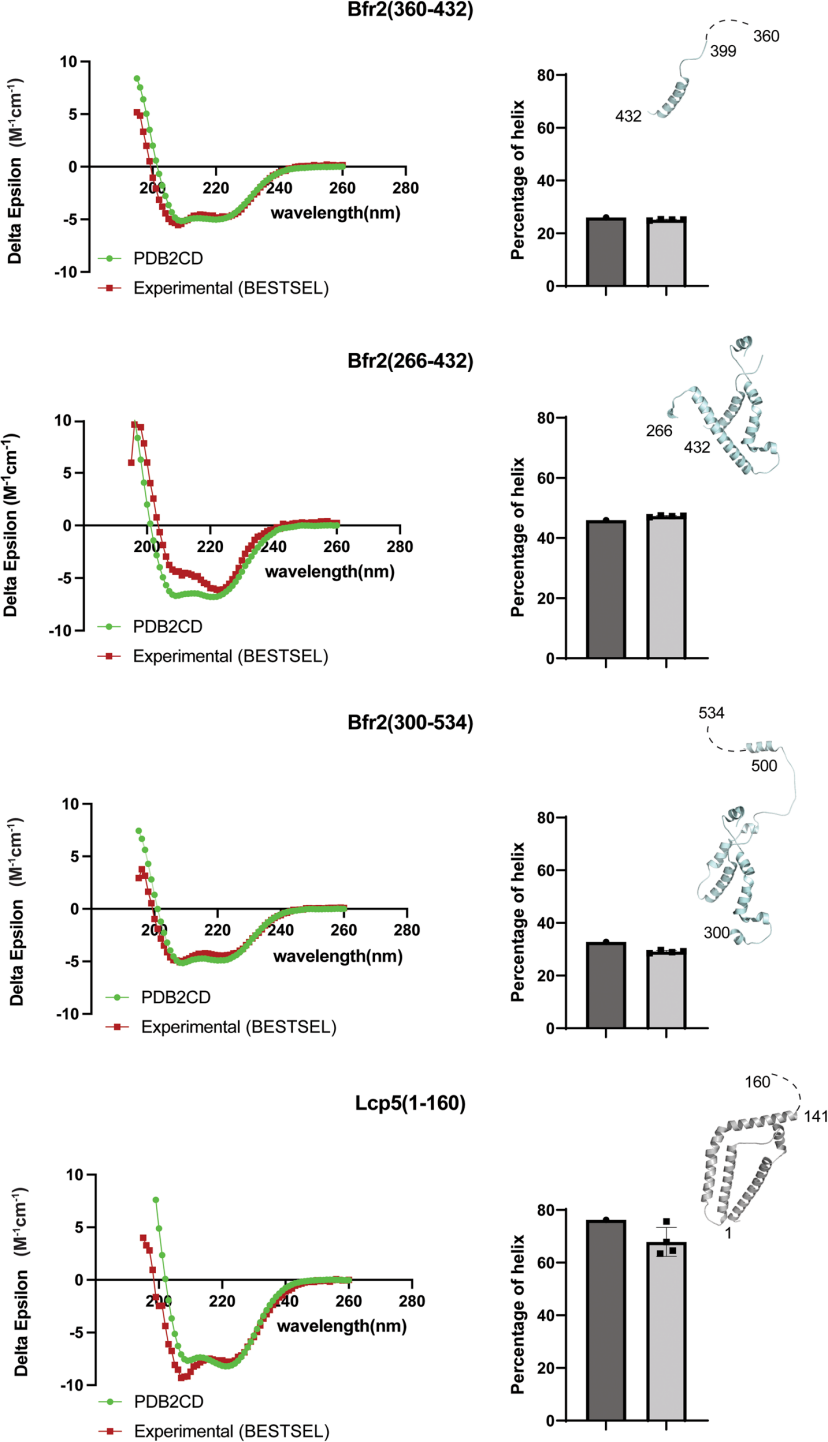
Circular Dichroism (CD) spectra of Bfr2 and Lcp5 fragments and data fitting. Left, the background-corrected Delta Epsilon (Δε) plots of fragments (red) collected at pH 7.4 in room temperature in comparison with those generated from the structures of fragments by PDB2CD (green). Right, comparison of the α-helix content for each protein fragment analyzed by BeStSel to that predicted from the structure of the corresponding protein fragment by PDB2CD. Error bars represent the experimental errors from three independent measurement of each protein fragment.

To provide experimental evidence for the cryoEM model of Bfr2 and Lcp5 in solution, we performed Circular Dichroism (CD) experiments with the three soluble MBP-fused Bfr2 fragments and the N-terminal domain of Lcp5. We also collected CD spectra of the isolated MBP that were used to correct those of the MBP-fused Bfr2 fragments (Fig. 2 and Supplementary Fig. 6). For all protein fragments, the ellipticity versus wavelength plots display peaks 193, 208 and 220 nm, characteristic of high helical content (Fig. 2 and Supplementary Fig. 6). We then compared the observed ellipticity versus wavelength plots to those generated based on the three-dimensional models of the Bfr2 fragments and Lcp5 N-terminal domain^31^ and the fitted alpha-helical contents to those predicted by PDB2CD^32^ based on our cryoEM model. The results show that the helical contents of the Bfr2 and Lcp5 structure are in good agreement with those measured by CD in solution. The slight upward shift at 208 nm for Bfr2 (266–432) between the PDB2CD-prediced and the measured ellipticity may reflect the structural changes of Bfr2 (266–432) in the present (predicted) and absent (measured) of Lcp5. It may also reflect aggregation of the Bfr2 fragments purified in E. coli (Supplementary Fig. 4a).

### The Bfr2-Lcp5 structure suggests a competition with Krr1 binding

**Fig. 3:**
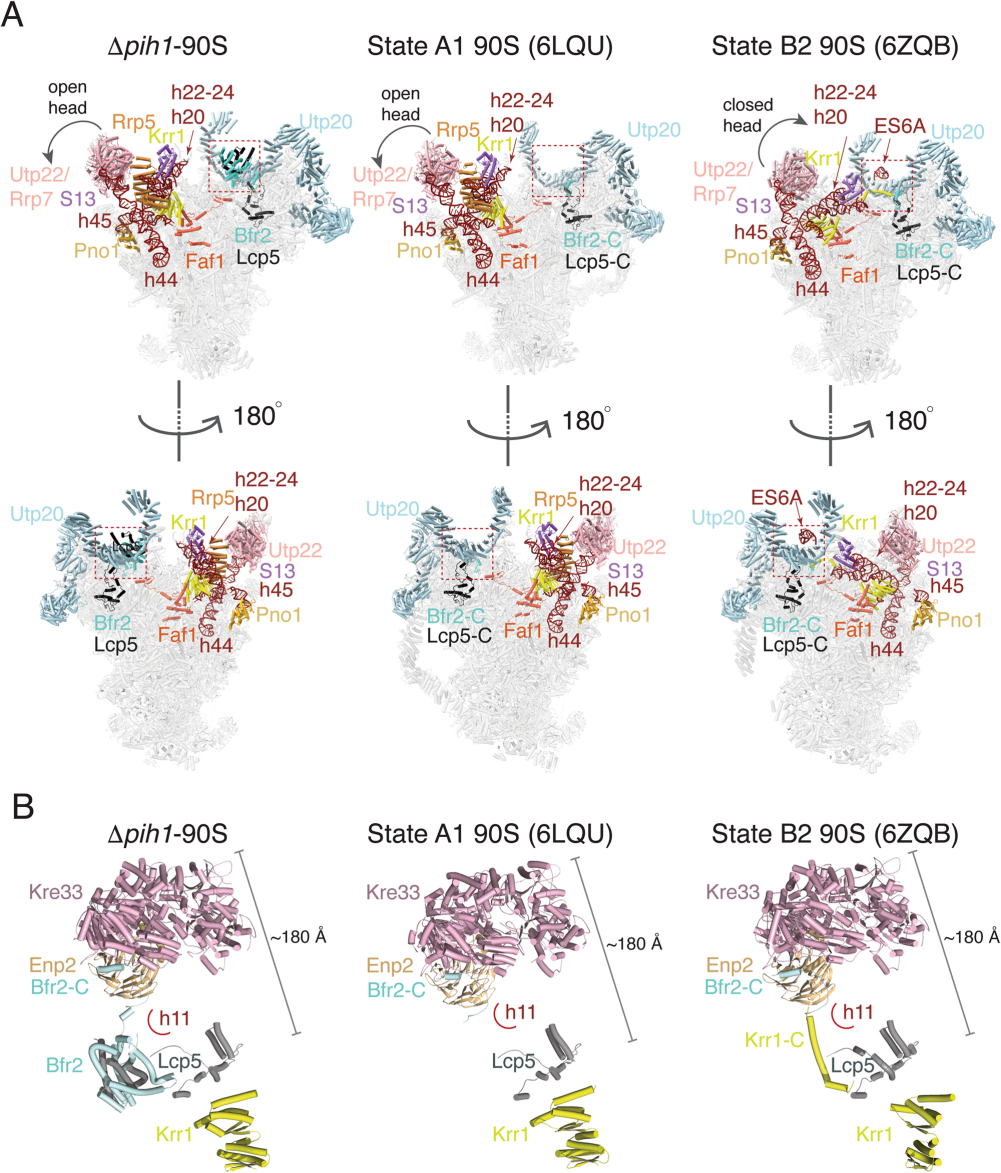
Comparison of Δ*pih1*-90S structure to two representative 90S structures. State A1 represents 90S in a storage state while State B2 represents a successive Krr1-bound state. **a** Overviews in two opposite orientations (upper and lower) of 90S structures with labeled key factors shown as colored ribbons. The boxed region indicates where Bfr2-Lcp5 is bound. Left, Δ*pih1*-90S, Middle, State A1 of 90S (PDB ID: 6LQU), Right, State B2 of 90S (PDB ID: 6ZQB). **b** Structure of the Kre33 module and Krr1 in the three states as shown in **a**. Each protein is colored and labeled. The 18S helix 11 (h11) is schematically shown as an arc. Bfr2-C and Krr1-C refer to the C-terminal end of each of the two proteins.

We subsequently compared the Bfr2-Lcp5 bound Δ*pih1*-90S structure to other 90S structures and found the largest difference in the central domain (Fig. 3a). In addition to the wild-type Pwp2-Kre33 purified 90S^10^, Δ*pih1*-90S also shares a homology to that of state A1 observed in 90S isolated from the Dhr1-depleted cells (PDB ID: 6LQU)^6^. Like state A1, the central domain of Δ*pih1*-90S is open with helical regions of 18S (h20, h22-h24) pointing up and stabilized by the associated proteins S13 and Rrp5 (Fig. 3a). In addition, the extended segment 6 A (ES6A) or the C-terminal tail of Krr1 is not formed in either complex (Fig. 3a). Different from state A1, however, Δ*pih1*-90S has the bound Bfr2-Lcp5 complex.

When superimposed with the state that contains the same set of assembly factors but successive (state B2 in the Noc4-Dhr1-purified 90S, PDB ID 6ZQB or state C in the Dhr2-depleted 90S, PDB ID 6LQQ), herein state B2, the Δ*pih1*-90S structure reveals a competition of Bfr2-Lcp5 in binding with the C-terminal tail of Krr1 (Fig. 3a). In state B2, the central domain is closed with pre-18S helices h20-h24 placed near the UTP20 C-terminal arc. The C-terminal tail of Krr1 is placed near h11 where Bfr2-Lcp5 complex is bound in Δ*pih1*-90S (Fig. 3a, b). Studies of the successive states following B2 or C in two differently purified 90S indicate that the release of the Krr1-Faf1 complex is required before the release of the Kre33-Enp2-Bfr2-Lcp5 module^6,7^. The stably bound Bfr2-Lcp5 complex in place of Krr1 C-terminal tail could disrupt this orderly transition in Δ*pih1*-90S.

The observed Bfr2-Lcp5 complex in the Δ*pih1*-90S provides a structure model of the Kre33 module previously incomplete (Fig. 3b). The C-terminal tail of Bfr2 (residues 454–500) wraps around the β-propeller of Enp2 while its central domain (residues 168–432) interacts with the N-terminal domain of Lcp5 (residues 1–142). Both Bfr2 and Lcp5 form an extended structure that mediates long-range interactions within the Kre33 module spanning 160 Å – 200 Å in distance (Fig. 3b).

## Discussion

We created the yeast strain that is depleted of the key R2TP component Pih1 (Δ*pih1*) and examined the structure of 90S isolated from the Δ*pih1*cells. R2TP is a maturation chaperone for the U3 snoRNP^19,33–35^. The U3 snoRNP is one of the nine modules incorporated into the 90S complex and plays a key role in 90S assembly and A1 cleavage. In absence of Pih1, which leads to slow production of U3 snoRNP, the Δ*pih1*-90S exhibits largely similar structure as that of 90S isolated from the wild-type cells under the similar nutrition-limiting condition^10^. However, the Δ*pih1*-90S structure showed strong enough density for the previously unobserved complex Bfr2-Lcp5 that allowed the determination of its structure with the aid of the recently available AlphaFold that employs an effective deep learning algorithm^25^. Although Pih1 does not associate with 90S or is connected to the Kre33 module directly, the fact that the Δ*pih1*-90S trapped Bfr2-Lcp5 complex suggests its possible role in facilitating 90S conformational transitions. The Bfr2-Lcp5 complex formed between the N-terminal domain of Lcp5 and the central domain of Bfr2 bridges an important interaction among the four proteins within the Kre33 module.

Interestingly, a stable Bfr2-Lcp5 complex has not been observed in any of the Kre33-moduel-containing states that are able to undergo transitions^6,7^. In these 90S structures, the departure of the Krr1-Faf1 module leads to disassociation of all members of the Kre33 module and rearrangement of the central domain in preparation for A1 cleavage. In the Δ*pih1*-90S structure, the Bfr2-Lcp5 complex is observed to occupy the same site as the C-terminal tail of Krr1 while the central domain is in an open conformation. It is thus possible that the Bfr2-Lcp5 complex plays a role in locking 90S in this form that prevents its progression under nutrient-limiting conditions by slowing the release of the Krr1-Faf1 complex. This is consistent with the observation that in the Noc4-Dhr1 purified 90S, a state (pre-A1) was observed where only Lcp5 but not Bfr2 was released^7^, suggesting a possibly weak or no interaction between Lcp5 and Bfr2 under normal 90S progression.

**Fig. 4:**
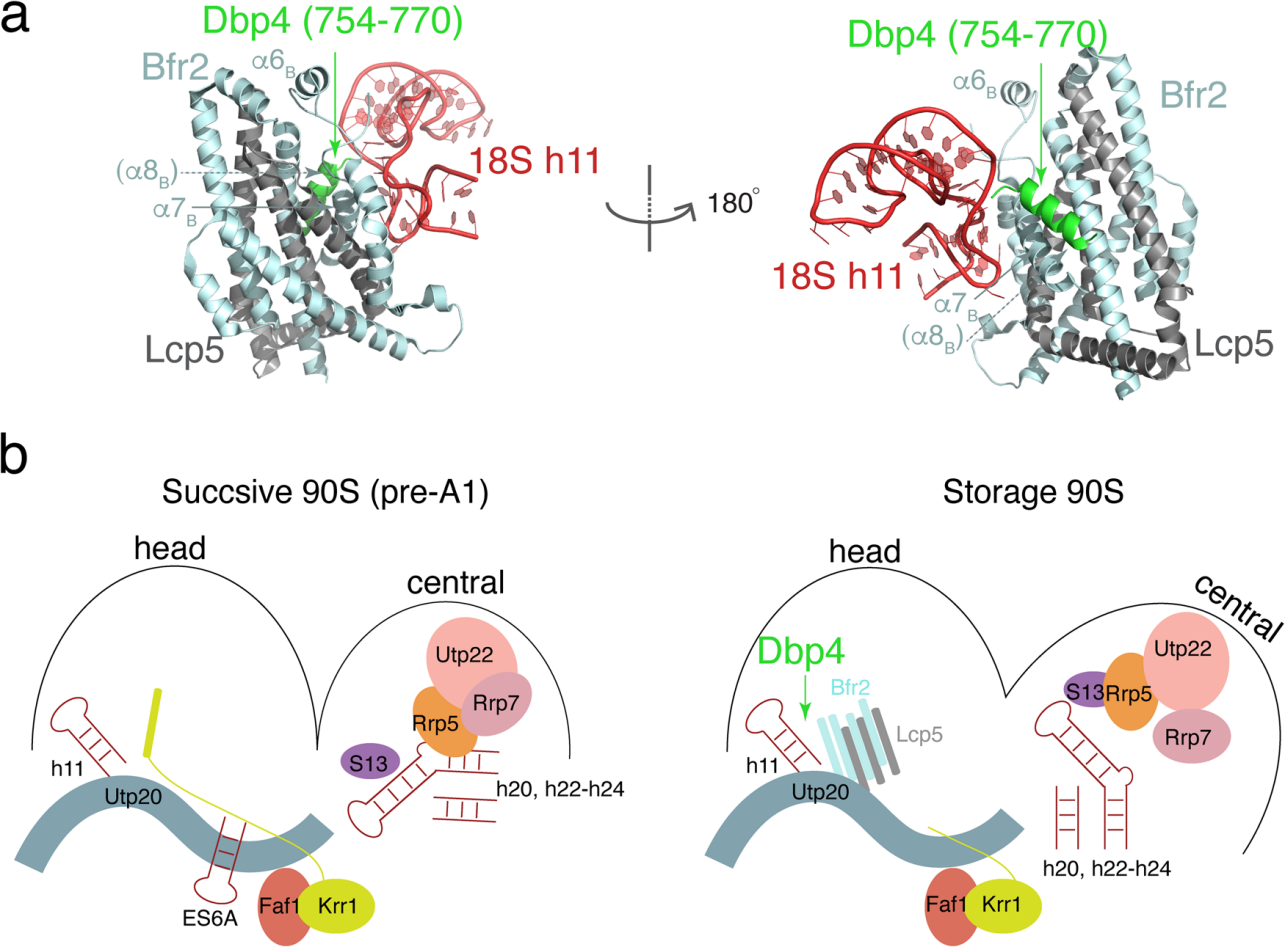
A proposed mechanism of Bfr2-Lcp5-mediated locking of 90S involving helicase Dbp4. **a** Structural model of Bfr2-Lcp5-Dbp4 complex in two orientations. The C-terminal helix of Dbp4 (residues 754–770) is colored in green while Bfr2 is in cyan and Lcp5 in gray, respectively. **b** Schematic conformational transitions between the successive 90S (left) and the 90S in the storage conformation involving Bfr2-Lcp5 and Dbp4. Removal or dissociation of Bfr2-Lcp5 complex by Dbp4 could trigger stabilization of the Krr1 C-terminal helix and closing of the central domain.

The DEAD-box protein Dbp4 was previously shown to interact with Bfr2 and Enp2^15^ and is required for the release of U14 snoRNA that guides 2’-O-methylation of the 5’ domain nucleotide 414^15,36^. Our modeled Bfr2-Lcp5 complex is near nucleotide 414 and thus, suggests that Dbp4 can transiently engage with 90S at a location near the 5’ domain. Taking advantage of the structural homology between Bfr2-Lcp5 and exosome factors Rrp6-Rrp47 and the known interaction between Rrp6-Rrp47 and the helicase Mtr4^30^, we modeled the Bfr2-Lcp5-Dbp4 complex by AlphaFold. In addition to the previously predicted Bfr2-Lcp5 complex, AlphaFold also placed the C-terminal tail of Dbp4 (residues 754–770) near α7 of Bfr2 in place of α8 (Fig. 4a) while maintaining the two well-conserved RecA domains. It is thus possible that a C-terminal tail of Dbp4 outside its two RecA domains forms a complex with Bfr2, similar to the binding of the N-terminal helix of Mtr4 to Rrp6-Rrp47^30^. Since our model is from a non-progressing 90S, Dbp4 may independently interact with Bfr2 during a stage when U14 snoRNA is released from its site of action to transition 90S between its storage form and those of successive (Fig. 4b). Confirmation of this model requires capturing 90S structures that is bound with Dbp4.

## Materials and methods

### Construction of yeast strains

The *pih1-Δ* (BY4742, *pih1*Δ::KanMX) haploid strains were generated using a standard PCR-based gene manipulation strategy. In brief, a KanMX cassette flanking with 50 bp of the 5’UTR and 3’UTR regions of the *pih1* ORF was PCR-amplified from the plasmid pFA6-kanMX4 and transformed into wild-type BY4742 cells by the lithium acetate method. The transformants were selected on YPD solid medium containing G418. Correctly replaced strains were confirmed by PCR and DNA sequencing of the *pih1* ORF.

The Pwp2-TAP/Kre33-TwinStrepII strain were created by fusing TwinStrepII tag to the C-terminus of Kre33. The Kre33 specific tagging cassette were PCR amplified using the plasmid pFA6a-TwinstrepII-HphMX6 as template and later transformed into Pwp2-TAP (BY4741, Pwp2-TAP::HisMX6) strain, purchased from Open Biosystem. Transformants were selected on YPD plates containing hygromycin-B. Correct fusion of the tag into the genome was confirmed by sequencing the *kre33* ORF.

To tag the mutant strains, heterozygous strains were created by crossing the BY4741 pwp2-TAP-his/kre33-TwinStrepII-hph haploid strain with the *pih1-Δ* BY4742 strain followed by sporulation. At least 20 tetrads were dissected on YPD agar plates from each sporulated heterozygous strain. Tetrad dissection plates were replica plated onto SD-HIS, YPD + G418, YPD + Hygromycin B plates for genotyping. Mating types of haploids were determined by crossing with tester strains. Appropriate haploids were then confirmed by PCR and DNA sequencing.

### Purification of 90S from Δpih1 cells

The *Δpih1*/pwp2-TAP/kre33-TwinStrep cells were grown at 30 °C in complete medium YPD (1% yeast extract, 2% peptone, 2% dextrose) to saturation (OD600 ≈ 16) in order to accumulate the processome as previously described^9^. The cells were harvested at 5000 rpm for 20 min, washed twice with cold ddH_2_O and once with cold ddH_2_O supplemented with Roche EDTA-free inhibitor cocktail and PMSF. Washed cell pellet was flash frozen with liquid nitrogen and cryogenically lysed by using a set of liquid nitrogen cooled mortar and pestle. Cryo-ground yeast powder was used for purification or stored at −80 °C.

To purify the SSU processome, yeast powder was hydrated and re-suspended with Buffer A (50 mM Tris, pH 7.7 at 22 °C, 150 mM NaCl, 1 mM EDTA, 0.1% Triton X-100, Roche EDTA-free protease inhibitor cocktail, 1 mM PMSF). Lysate was then clarified by centrifugation at 40,000 g for 10 min and the resulting supernatant was incubated with IgG Sepharose beads (GE healthcare) for one hour followed by washing with Buffer A without Triton X-100. The bound 90S was eluted from the IgG beads by overnight TEV protease cleavage at 4 °C and were further subjected to a second affinity purification with StrepTactin beads in Buffer B (50 mM Tris, pH 7.7 at 22 °C, 150 mM NaCl, 1 mM EDTA) according to manufacturer’s instruction. The 90S was eluted with Buffer B supplemented with 5 mM desthiobiotin and immediately used for EM experiments or stored at −80 °C for subsequent analysis.

### Mass spectroscopy analysis of the 90S from Δpih1/Enp1-TAP cells

Purified 90S was subjected to mass spectrometry analysis for protein identifications. Trypsin digested 90S sample was injected for nLC-MS/MS analysis (Easy Nano LC II system and a Velos LTQ-Orbitrap Mass Spectrometer, Thermo Scientific). Peptides were separated with a 10 cm × 75 µm C18AQ analytical column (Thermo Scientific). Peptide/protein identification was conducted by searching against a Uniprot *S. Cerevisiae* database with Proteome Discoverer 1.4 (Thermo Scientific) Sequest HT search engine (University of Washington). The precursor ions mass error tolerance is <5 ppm and fragment ions mass error tolerance is <0.8 Da and Percolator at a 1% FDR. Search parameters were as follows: trypsin, allowing 4 miscleavages, with oxidized methionine and carbamidomethyl cysteine as dynamic modifications. High confidence proteins are listed in Supplementary Data 1.

### CryoEM sample preparation, data collection, and structural determination

5 μl of the double-affinity tag purified sample with ~2 units UV260 nm absorbance was applied to a plasma cleaned glow discharged Quantifoil 2/2 grids (Quantifoil, Germany) that were pre-coated with a homemade layer of amorphous continuous carbon. The grids were blotted for 3 s at 4 °C in 100% humidity and plunged into liquefied ethane using Vitrobot MK IV (FEI, Hillsboro OR). The grids were stored in liquid nitrogen and later transferred into a Titan Krios (Thermo Fisher) electron microscope equipped with the BioQuantum K3 Imaging filter (Gatan) that operated at 300 kV. Micrographs were collected using 20 eV slit with Leginon^37^ at a nominal magnification of 81,000X in a super-resolution counting mode to a calibrated pixel size of 0.53 Å/pixel and with a defocus ranging of −1.3 to −2.5 microns. A total dose of 40 e-/Å^2^ was spread over fifty frames. A total of 4644 micrographs were collected, and after manual inspection, reduced to a final of 3477 with sufficient quality. Image processing was carried out in Relion 3.1^38^ unless otherwise stated. Frame alignment and dose compensation were carried out by the UCSF Motincor2^39^ where each pixel was binned by 2 into a pixel size of 1.074 Å/pixel. The Contrast transfer function (CTF) parameter was estimated by Gctf^40^. The frame-aligned images were randomly split into two halves (2320 and 1157, respectively). In total, 150,487 and 83,481 particles were picked using the LoG-based auto picking from the 2320 and 1157 micrographs, respectively. Initial Euler angles were determined via 3D classifications using an 80 Å low-pass filtered 90S map to avoid any model bias. The maps not resembling 90S were discarded which further reduced the particles to 199,534 particles. The resulted particles were refined to an overall resolution of 5.04 Å that was further improved to 3.99 Å after performing multiple rounds of CTF refinement. However, the maps showed the poor or diffused head and central domain. To improve the head domain density, the particles were classified into five classes by applying a 3D custom mask at the head domain region. The classes showing poor or incomplete head domain region were discarded and only the class showing good and complete head domain region (84,574 particles) were chosen and further refined using a 3D custom mask at the head domain region yielding the resolution of 5.04 Å with better head domain region. Similarly, to improve the central region, the focus refinement was performed using a 3D custom mask around the central region improving the map around the central region with a resolution of 7.24 Å. The FSC was estimated at 0.143-cutoff and the local resolution was estimated using Relion 3.1.

### AlphaFold prediction of protein models

The amino acids sequence of Bfr2, Lcp5 and Dbp4 were retrieved from Saccharomyces Genome Database (SGD) in FASTA format. Monomer models for Bfr2 and Lcp5 were predicted using AlphaFold v2.0.1 Open source code downloaded from github^24^. For the multimer models, either full-length or truncated protein sequences were connected by 14×(Glycine-Glycine-Serine) linker before being predicted as single proteins as described^26^.

### Purification of recombinant Bfr2 and Lcp5

The DNA sequences encoding Bfr2 or various fragments (1–534, 266–432, 360–432, 300–534) were PCR amplified from genomic DNA of BY4741 strain and cloned into the pMCSG19 vector using ligation independent cloning (LIC) along with that encoding Maltose Binding Protein (MBP) so they express MBP-fused Bfr2. The DNA encoding Lcp5(1–160) was PCR amplified similarly and cloned into vector pMCSG7 without an affinity tag using Gibson Assembly Cloning. To produce recombinant proteins, plasmids were freshly transformed into *E. coli* Rosetta™(DE3) competent cells (Novagen) and plated on LB plates containing ampicillin (50 μg/mL) and chloramphenicol (50 μg/mL). Single colonies were picked and grown overnight in liquid LB medium containing ampicillin and chloramphenicol as pre-cultures. Pre-cultures were used to inoculate 1 liter of LB that were grown at 37 °C until OD600 reached 0.6–0.8 before being induced with 0.2 mM isopropyl β-D-1-thiogalactopyranoside (IPTG). The cells were allowed to grow at 16 °C overnight before being harvested at 4 °C by pelleting at 6000 × *g* for 15 min in a Beckman Coulter Avanti J-20 XPI centrifuge (OPTIMA). The cell pellets were suspended in buffer A (25 mM Tris, pH 7.5, 500 mM NaCl, and 5% glycerol) and lysed by sonication. Supernatant was loaded to an amylose affinity column equilibrated with 5 column volumes of buffer A. Following washing with buffer A, the protein samples were eluted with buffer B (25 mM Tris, pH 7.5, 500 mM NaCl, 10 mM maltose and 5% glycerol). The elutant was further purified by ion exchange chromatography with a monoQ (GE Health Sciences) followed by gel filtration on a Superdex Increase 200 10/300 GL column (GE Health Sciences). The protein samples were concentrated to 10–15 mg/ml and stored in −80 °C.

To analyze MBP-Bfr2(266–432)-Lcp5 interactions, cells expressing MBP-Bfr2(266–432) or Lcp5(1–160) were grown and harvested as either the mixture of both or individually. Cells expressing the two individual proteins and those expressing both were processed in parallel for loading and eluting from an amylose column.

### Circular Dichroism spectroscopy

All protein samples were dialysed in 1X Phosphate-buffered saline (PBS) buffer overnight before *Circular Dichroism* (CD) spectroscopy analysis. Typically, protein samples at 0.01 mg/ml in a quartz cuvette (Starna Cells) of 1.0 cm path length was placed into AVIV 410 spectrometer for CD spectrum acquisition at room temperature (25 °C). Each CD spectrum consisting of the absorbance values was acquired over a wavelength range of 195–260 nm. Each protein sample was measured in three independent scans and the final spectrum is the average of the three scans. To remove absorption background (buffer), three independent scans were made on the 1X PBS buffer. To remove signals from the fused Maltose binding protein (MBP), spectra of independently purified MBP in the same buffer were also acquired that were then subtracted from those of the MBP-Bfr2 fragments. All CD data were expressed as mean residue molar ellipticity in deg × cm2/dmol. The secondary structure content of the proteins was estimated by the deconvolution of far-UV CD spectra according to BeStSel^31^. PDB2CD^32^ was used to convert cryoEM models to CD spectrum data.

### Reporting summary

Further information on research design is available in the Nature Research Reporting Summary linked to this article.

## Supplementary information

Supplementary Information

Description of Additional Supplementary Files

Supplementary Data 1

Reporting Summary

## Data Availability

The atomic coordinates and associated density maps have deposited at Protein Data Bank with accession codes 7SUK & EMD-25441 for the Δ*pih1*-90S.

## References

[1] Klinge, S. & Woolford J. L. Jr. Ribosome assembly coming into focus. *Nat. Rev. Mol. Cell Biol.* **20**, 116–131 (2019). 10.1038/s41580-018-0078-y PMC7725133 30467428

[2] Woolford JL, Baserga SJ (2013). Ribosome biogenesis in the yeast Saccharomyces cerevisiae. Genetics.

[3] Pena C, Hurt E, Panse VG (2017). Eukaryotic ribosome assembly, transport and quality control. Nat. Struct. Mol. Biol..

[4] de la Cruz J, Karbstein K, Woolford JL (2015). Functions of ribosomal proteins in assembly of eukaryotic ribosomes in vivo. Annu. Rev. Biochem..

[5] Yelick PC, Trainor PA (2015). Ribosomopathies: global process, tissue specific defects. Rare Dis..

[6] Du Y (2020). Cryo-EM structure of 90S small ribosomal subunit precursors in transition states. Science.

[7] Cheng J (2020). 90 S pre-ribosome transformation into the primordial 40 S subunit. Science.

[8] Sun Q. et al. Molecular architecture of the 90 S small subunit pre-ribosome. *eLife* **6**, e29876 (2017). 10.7554/eLife.22086 PMC5354517 28244370

[9] Chaker-Margot M., Barandun J., Hunziker M. & Klinge, S. Architecture of the yeast small subunit processome. *Science* **355**, eaal1880 (2017). 10.1126/science.aal1880 27980088

[10] Barandun J. et al. The complete structure of the small-subunit processome. Nat. Structural Mol. Biol. **24**, 944–953 (2017). 10.1038/nsmb.3472 PMC12919394 28945246

[11] Cheng J (2019). Thermophile 90 S pre-ribosome structures reveal the reverse order of co-transcriptional 18S rRNA subdomain integration. Mol. Cell.

[12] Perez-Fernandez J, Roman A, De Las Rivas J, Bustelo XR, Dosil M (2007). The 90 S preribosome is a multimodular structure that is assembled through a hierarchical mechanism. Mol. Cell. Biol..

[13] Chaker-Margot M, Hunziker M, Barandun J, Dill BD, Klinge S (2015). Stage-specific assembly events of the 6-MDa small-subunit processome initiate eukaryotic ribosome biogenesis. Nat. Struct. Mol. Biol..

[14] Zhang L, Wu C, Cai G, Chen S, Ye K (2016). Stepwise and dynamic assembly of the earliest precursors of small ribosomal subunits in yeast. Genes Dev..

[15] Soltanieh S, Lapensee M, Dragon F (2014). Nucleolar proteins Bfr2 and Enp2 interact with DEAD-box RNA helicase Dbp4 in two different complexes. Nucleic Acids Res..

[16] Cheng J., Kellner N., Berninghausen O. & Hurt E. Beckmann R. 3.2-A-resolution structure of the 90 S preribosome before A1 pre-rRNA cleavage. *Nat. Structural Mol. Biol.* **24**, 954–964 (2017). 10.1038/nsmb.3476 28967883

[17] Lafontaine DL, Tollervey D (2000). Synthesis and assembly of the box C + D small nucleolar RNPs. Mol. Cell. Biol..

[18] Yu G, Zhao Y, Li H (2018). The multistructural forms of box C/D ribonucleoprotein particles. RNA.

[19] Zhao R (2008). Molecular chaperone Hsp90 stabilizes Pih1/Nop17 to maintain R2TP complex activity that regulates snoRNA accumulation. J. Cell Biol..

[20] Gonzales FA, Zanchin NI, Luz JS, Oliveira CC (2005). Characterization of Saccharomyces cerevisiae Nop17p, a novel Nop58p-interacting protein that is involved in Pre-rRNA processing. J. Mol. Biol..

[21] McKeegan KS, Debieux CM, Watkins NJ (2009). Evidence that the AAA+ proteins TIP48 and TIP49 bridge interactions between 15.5K and the related NOP56 and NOP58 proteins during box C/D snoRNP biogenesis. Mol. Cell. Biol..

[22] Kakihara Y, Makhnevych T, Zhao L, Tang W, Houry WA (2014). Nutritional status modulates box C/D snoRNP biogenesis by regulated subcellular relocalization of the R2TP complex. Genome Biol..

[23] Prieto MB, Georg RC, Gonzales-Zubiate FA, Luz JS, Oliveira CC (2015). Nop17 is a key R2TP factor for the assembly and maturation of box C/D snoRNP complex. BMC Mol. Biol..

[24] Quinternet M (2015). Structure/function analysis of protein–protein interactions developed by the Yeast Pih1 platform protein and its partners in Box C/D snoRNP Assembly. J. Mol. Biol..

[25] Jumper J (2021). Highly accurate protein structure prediction with AlphaFold. Nature.

[26] Bammert L, Jonas S, Ungricht R, Kutay U (2016). Human AATF/Che-1 forms a nucleolar protein complex with NGDN and NOL10 required for 40S ribosomal subunit synthesis. Nucleic Acids Res..

[27] Yu G (2019). Yeast R2TP interacts with extended termini of client protein Nop58p. Sci. Rep..

[28] Evans R. et al. Protein complex prediction with AlphaFold-Multimer. *bioRxiv* (2021).

[29] Humphreys I. R. et al. Computed structures of core eukaryotic protein complexes. *Science* **374**, eabm4805 (2021). 10.1126/science.abm4805 PMC7612107 34762488

[30] Schuch B (2014). The exosome-binding factors Rrp6 and Rrp47 form a composite surface for recruiting the Mtr4 helicase. EMBO J..

[31] Micsonai A (2015). Accurate secondary structure prediction and fold recognition for circular dichroism spectroscopy. Proc. Natl Acad. Sci. USA.

[32] Mavridis L, Janes RW (2017). PDB2CD: a web-based application for the generation of circular dichroism spectra from protein atomic coordinates. Bioinformatics.

[33] Zhao R (2005). Navigating the chaperone network: an integrative map of physical and genetic interactions mediated by the hsp90 chaperone. Cell.

[34] Kakihara Y, Houry WA (2012). The R2TP complex: discovery and functions. Biochimica et. Biophysica Acta.

[35] Boulon S (2008). The Hsp90 chaperone controls the biogenesis of L7Ae RNPs through conserved machinery. J. Cell Biol..

[36] Kos M, Tollervey D (2005). The putative RNA helicase Dbp4p is required for release of the U14 snoRNA from preribosomes in saccharomyces cerevisiae. Mol. Cell.

[37] Suloway C (2005). Automated molecular microscopy: the new Leginon system. J. Struct. Biol..

[38] Zivanov J. et al. New tools for automated high-resolution cryo-EM structure determination in RELION-3. *eLife* **7**, e42166 (2018). 10.7554/eLife.42166 PMC6250425 30412051

[39] Zheng SQ (2017). MotionCor2: anisotropic correction of beam-induced motion for improved cryo-electron microscopy. Nat. methods.

[40] Zhang K (2016). Gctf: Real-time CTF determination and correction. J. Struct. Biol..

