# Supplementary material for: Artificial intelligence-assisted cryoEM structure of Bfr2-Lcp5 complex observed in the yeast small subunit processome
Source: Commun Biol. 2022 Jun 1;5:523. doi: 10.1038/s42003-022-03500-y (PMC9160021; doi:10.1038/s42003-022-03500-y)
Supplement: Supplementary file 1 — Supplementary Information [file 42003_2022_3500_MOESM1_ESM.pdf]

Supplementary Materials for

Artificial Intelligence-assisted cryoEM Structure of Bfr2-Lcp5 Complex Observed in the Yeast Small Subunit Processome

Yu Zhao[1], Jay Rai[1], Chong Xu[2], and Hong Li[1,2]*

[1]Institute of Molecular Biophysics, Florida State University, Tallahassee, FL 32306, USA.

[2]Department of Chemistry and Biochemistry, Florida State University, Tallahassee, FL 32306, USA.

*Corresponding author: hong.li@fsu.edu

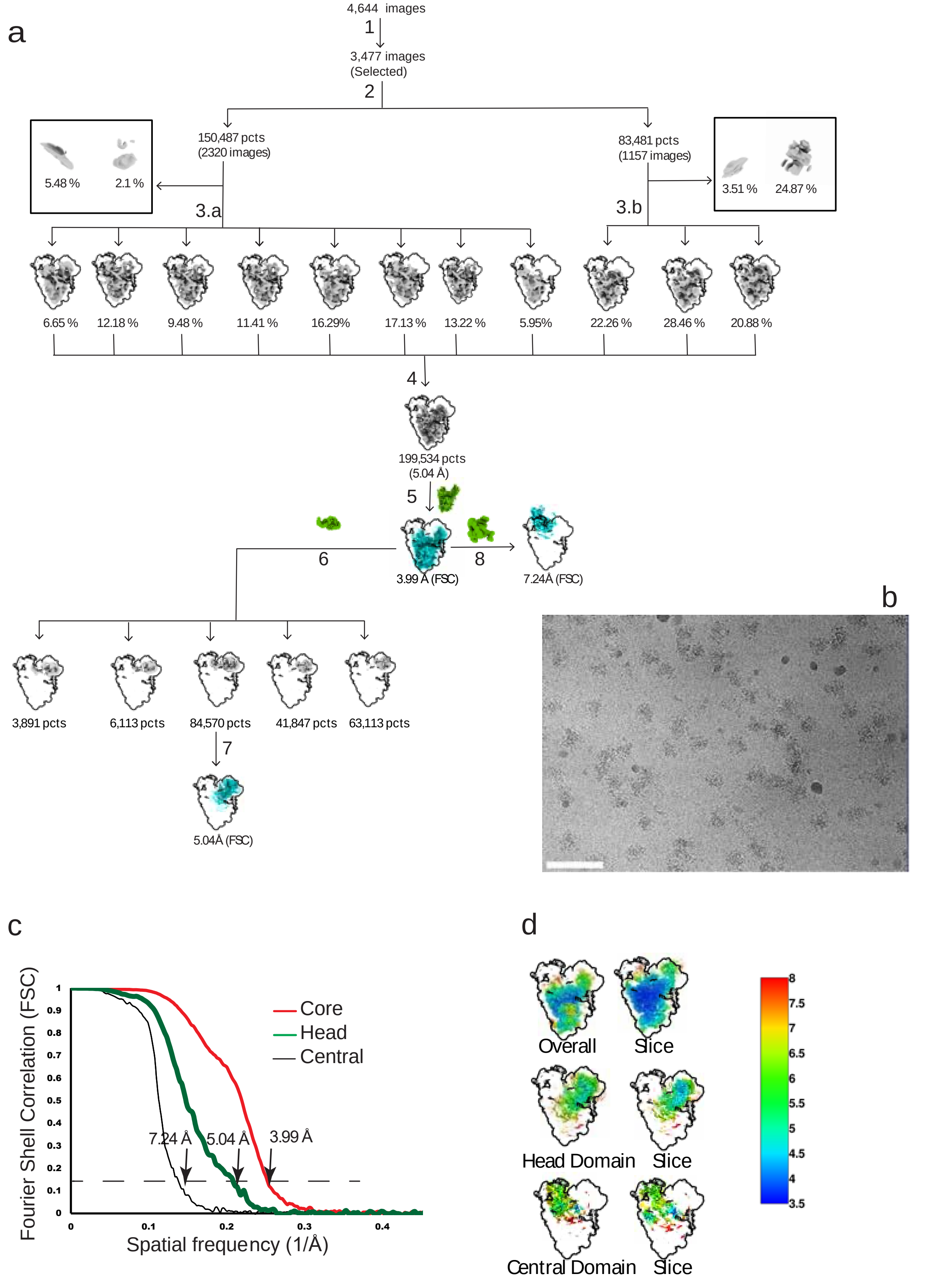

Supplementary Figure 1.

Supplementary Figure 1, related to Figures 1 & 2. Flow chart of cryoEM structure determination and analysis of *Δpih1*-90S. **a.** Image processing, particle selection, and reconstruction schemes. The masks used in focused classification and refinement are shown in green. The final maps used for model building and refinement are shown in teal. A final of 3,477 images were selected for further processing as two batches (1) that led to a total of 233,968 particles (pcts) (2). The first half contained 150,487 pcts that were classified to 10 classes (3.a). The other half contained 83,481 pcts that were classified into 5 classes (3.b). Particles with bad features were removed, which led to the combined 199,534 pcts (4). Focused refinement with the mask around the core or the central domain led to two maps with 3.99Å and 7.24 Å resolution, respectively (5, 8). To improve resolution for the head (5′ domain), particles were further classified without alignment into five classes using a 3D custom mask on the head domain (6). The classes with good head density (84,570 pcts) were refined using the custom3D mask, which resulted in an overall resolution of 5.04 Å (7). **b.** A representative raw micrograph (scale bar 200 nm). **c.** The Fourier Shell Correlation (FSC) curves of the core, head and central domain, respectively, 0.143 cutoff was used for resolution estimation. **d.** Local resolution estimated by Resmap showing both low- and high-resolution regions.

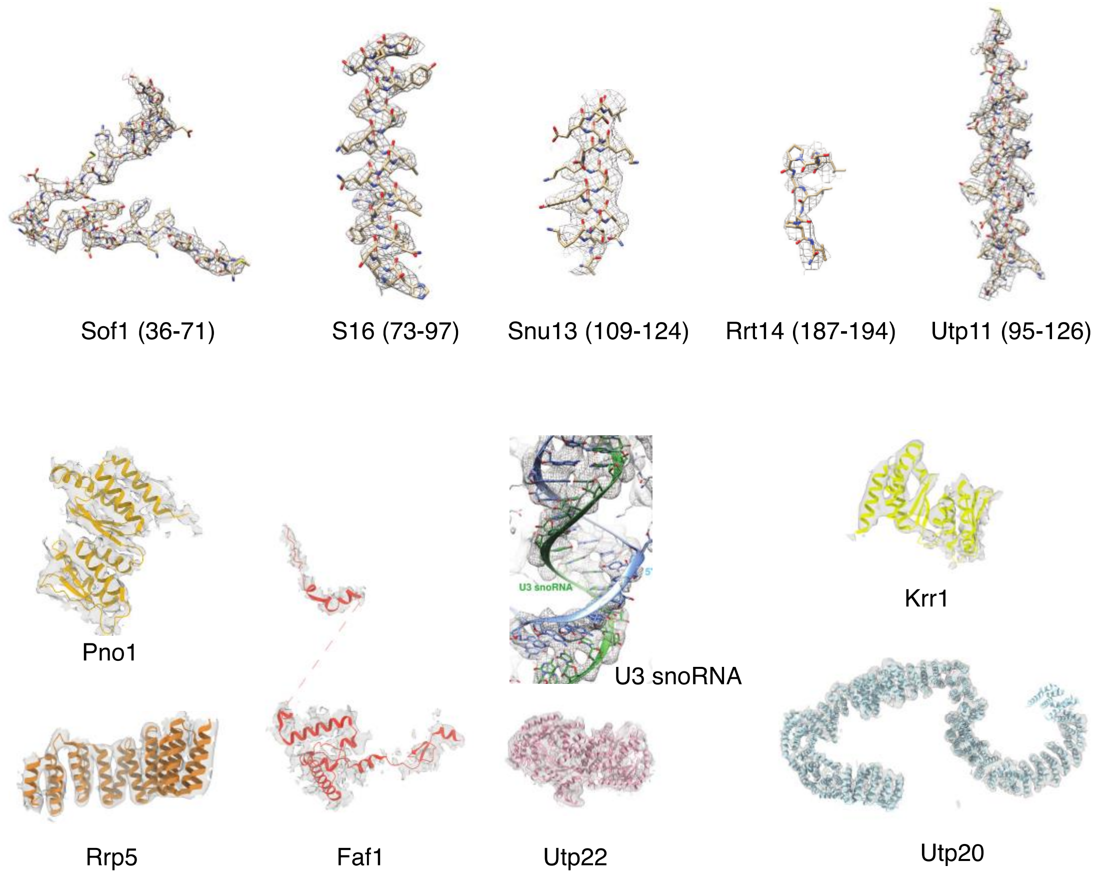

Supplementary Figure 2, related to Figures 1 & 2. Electron potential density for selected regions of the *Δpih1*-90S. Names of the subunits and the residue ranges are labeled.

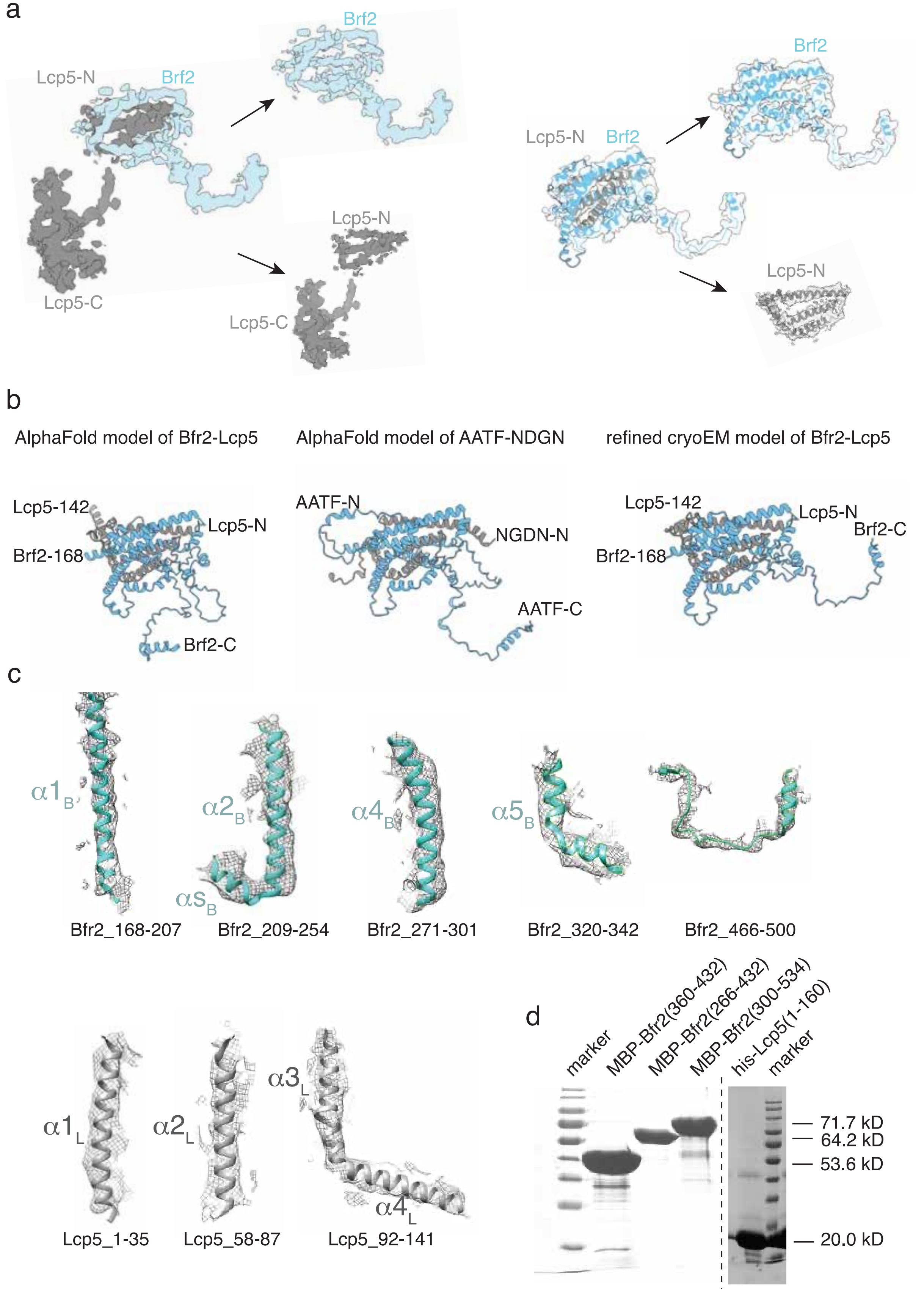

Supplementary Figure 3.

Supplementary Figure 3, related to Figures 1-3. Overview of model building process for Bfr2-Lcp5 complex. **a.** Left, electron potential density identified for Bfr2 (cyan) and Lcp5 (gray), respectively. Right, the same electron potential density shown on the left superimposed with the final Bfr2-Lcp5 model. **b.** Comparison of AlphaFold predicted Bfr2-Lcp5 (left), AlphaFold predicted human homolog of Brf2-Lcp5, AATF-NGDN (middle), and the cryoEM structure of Bfr2-Lcp5 (right). Locations of the termini of the models are indicated. **c.** Close-up views of fitting of a-helices of Bfr2 (teal) and Lcp5 (gray) to the assigned electron potential density (mash). The secondary structure labels are the same as those in Figure 1b. **d.** SDS-PAGE analysis of purified MBP-Bfr2 and His-Lcp5 fragments along with protein molecular weight standards.

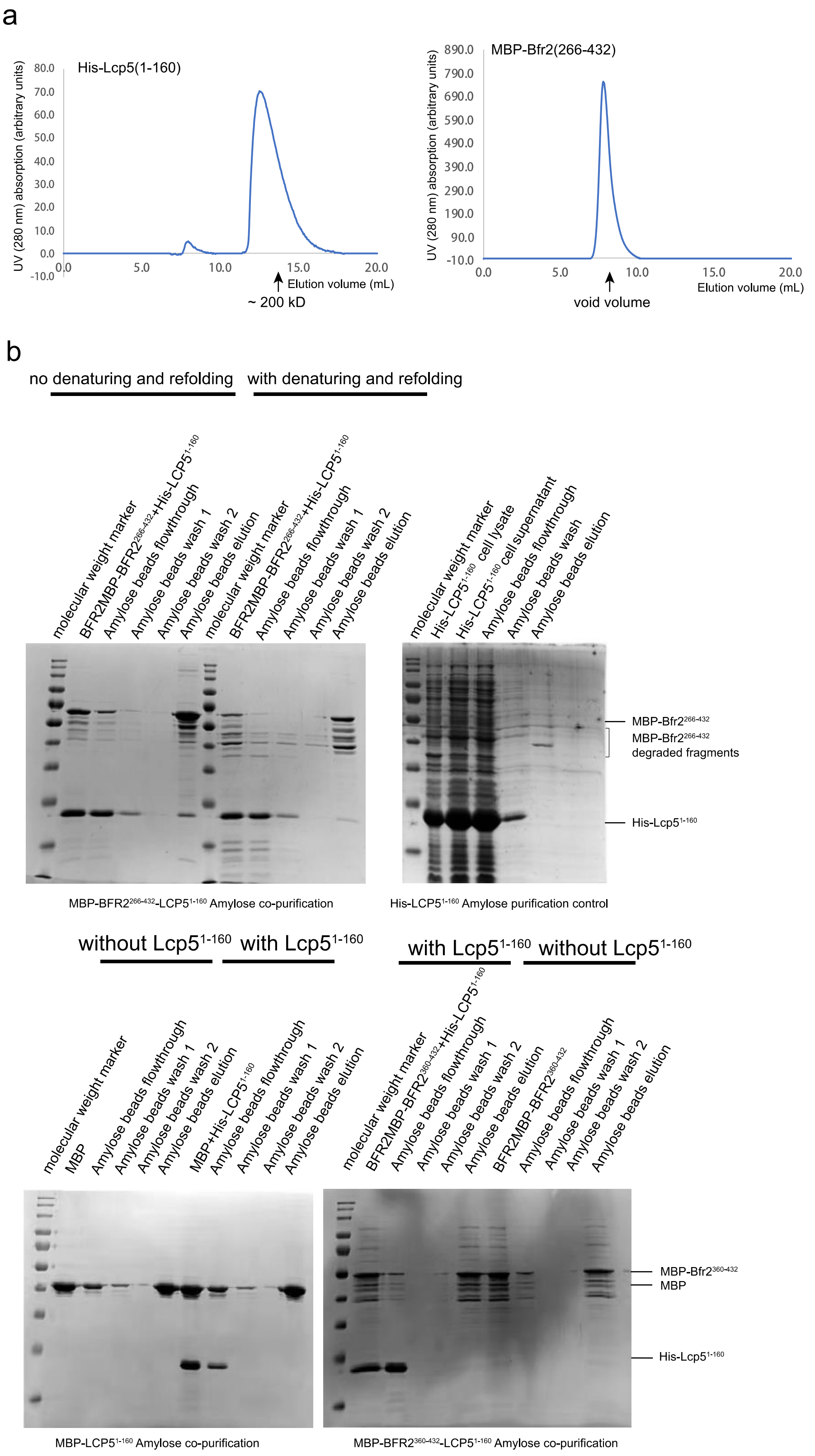

Supplementary Figure 4.

Supplementary Figure 4, related to Figures 1 and 2. Characterization of purified MBP-Bfr2(266-432) and His-Lcp5(1-160) interactions by co-purification. **a.** Gel filtration profiles of MBP-Bfr2(266-432) (right) and His-Lcp5(1-160) (left). **b.** Top, co-purification of MBP-Bfr2(266-432) and His-Lcp5(1-160) on an amylose column with no or with urea-mediated refolding (top left). For urea denaturing and refolding, the two proteins were denatured in 8 M urea followed by refolding while slowly dialyzing urea away. The refolded sample was then subjected to co-purification. Purification of His-Lcp5(1-160) on the amylose column was used as a control (top right). Lower, co-purification of MBP (lower left), MBP-Bfr2(360-432) (lower right) and His-Lcp5(1-160) on an amylose column.

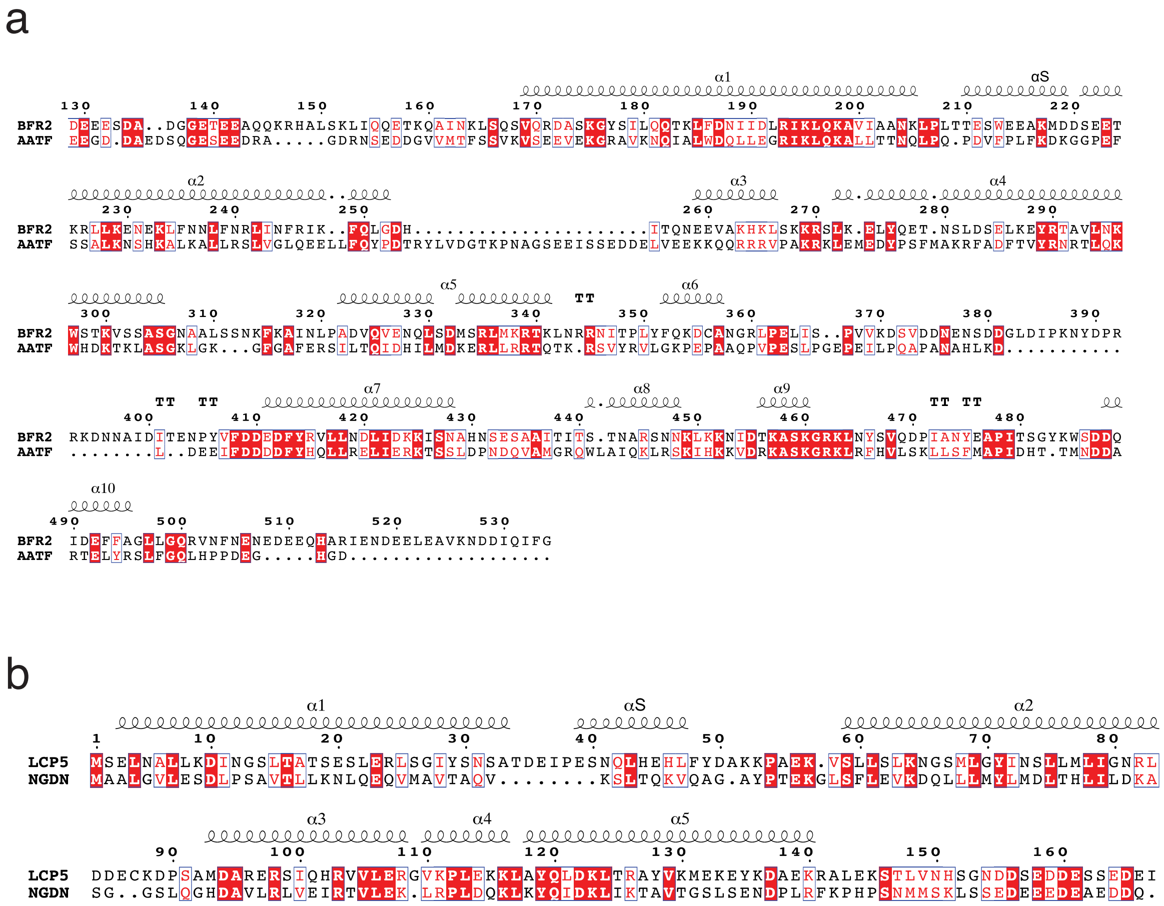

Supplementary Figure 5.

Supplementary Figure 5, related to Figures 1-4. Sequence alignment between yeast and human homologs. **a.** Sequence alignment between Bfr2 and human homology AATF with secondary structural elements superimposed. **b.** Sequence alignment between Lcp5 1-141 and human homology NGDN with secondary structural elements superimposed.

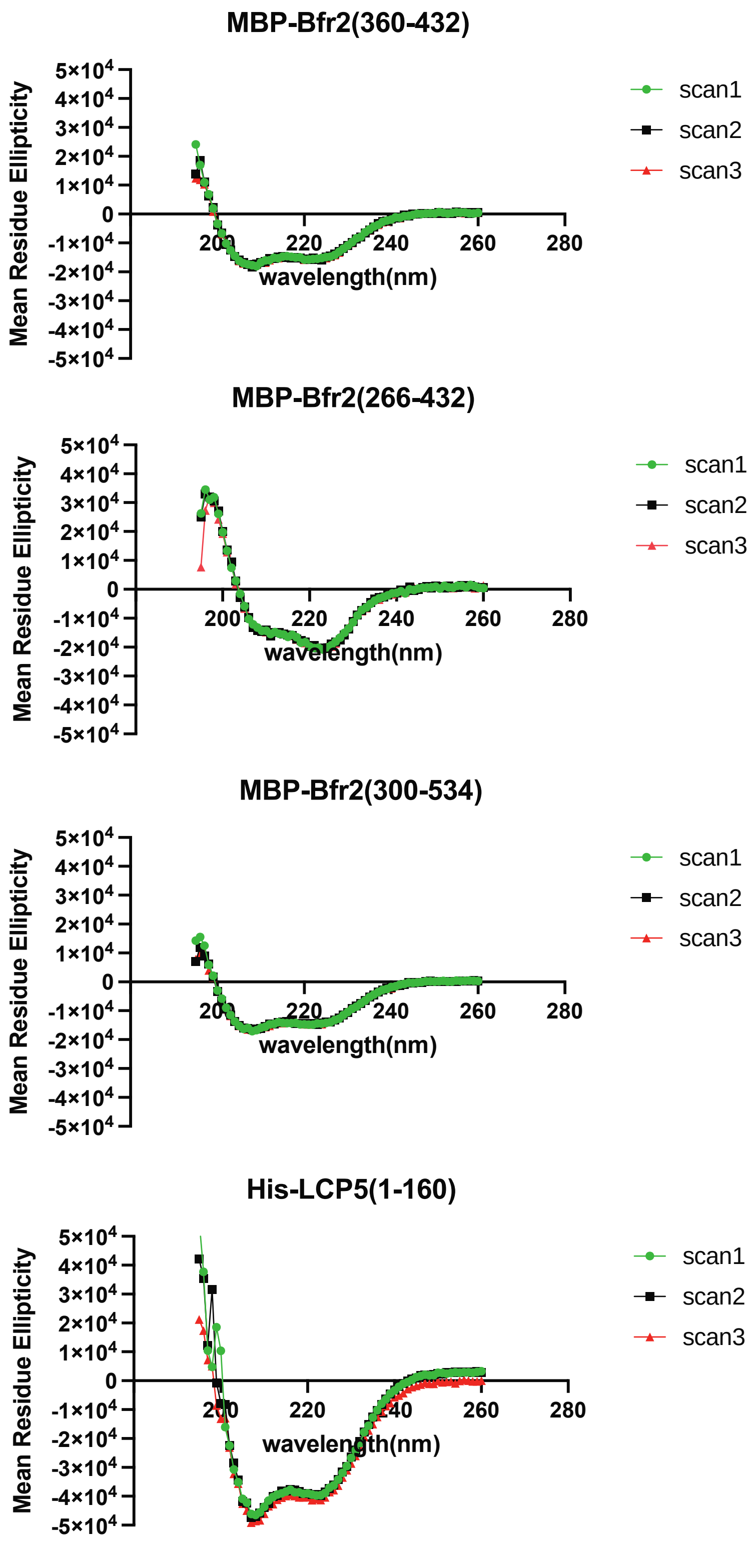

Supplementary Figure 6.

Supplementary Figure 6, related to Figure 2. Raw scans of Circular Dichroism (CD) spectra of Bfr2 and Lcp5 fragments in triplicates without correcting MBP signals. Three scans of the Delta Episilon plots of Bfr2 and Lcp5 fragments were collected at pH 7.4 at room temperature.
