# Supplementary material for: Artificial intelligence-assisted cryoEM structure of Bfr2-Lcp5 complex observed in the yeast small subunit processome
Source: Commun Biol. 2022 Jun 1;5:523. doi: 10.1038/s42003-022-03500-y (PMC9160021; doi:10.1038/s42003-022-03500-y)
Supplement: Supplementary file 2 — Description of Additional Supplementary Files [file 42003_2022_3500_MOESM2_ESM.pdf]

Description of Additional Supplementary Files

File name: Supplementary Data 1
Description: List of Mass Spectrometry Identified Proteins from Isolated Dpih1-90S.
